# Enhanced photocatalytic performance of milkvetch-derived biochar via ZnO–Ce nanoparticle decoration for reactive blue 19 dye removal

**DOI:** 10.1038/s41598-023-45145-9

**Published:** 2023-10-19

**Authors:** Fatemeh Jahani, Basir Maleki, Mohsen Mansouri, Zahra Noorimotlagh, Seyyed Abbas Mirzaee

**Affiliations:** 1 Department of Chemical Engineering, Faculty of Engineering, Ilam University, Ilam, Iran; 2 Health and Environment Research Center, Ilam University of Medical Sciences, Ilam, Iran; 3 Department of Environmental Health Engineering, School of Health, Ilam University of Medical Sciences, Ilam, Iran

**Keywords:** Environmental chemistry, Nanoparticles

## Abstract

In this research, the photocatalytic removal of reactive blue 19 (RB19) dye is investigated employing zinc oxide/cerium (ZnO@Ce) nanoparticles decorated with biochar under LED irradiation. Synthesis of ZnO@Ce nanoparticles decorated with biochar was performed utilizing the co-precipitation procedure and, then, the texture and morphology of the fabricated nanocomposite were analyzed using energy dispersive X-ray (EDX), field emission scanning electron microscopy (FE-SEM), X-ray powder diffraction (XRD), transmission electron microscopy (TEM), Brunauer–Emmett–Teller (BET), and Fourier transform infrared (FTIR) spectroscopy techniques. Moreover, FE-SEM images demonstrate that ZnO–Ce nanoparticles were successfully decorated on the surface of biochar. The specific surface areas of biochar and biochar/ZnO–Ce were 519.75 and 636.52 m^2^/g, respectively. To achieve the maximum yield in the removal of RB19 dye, the effects of operating variables including dye concentration, LED lamp power, biochar@ZnO–Ce catalyst dose, pH and H_2_O_2_ dose were explored. Besides, the maximum percentage of RB19 dye removal was 96.47% under optimal conditions, i.e. catalyst dosage of 100 mg, H_2_O_2_ dosage of 1 mL, pH of 9, initial dye concentration of 5 ppm, LED power of 50 W, and reaction time of 140 min. Furthermore, the kinetic analysis reveals that the removal of RB19 dye follows the pseudo-first order kinetic model, with calculated values of a reaction rate constant of 0.045 min^−1^ and a correlation coefficient of R^2^ = 0.99, respectively. Moreover, the reusability and recyclability of biochar@ZnO/Ce nanocatalyst was promising over five runs, with only a 6.08% decrease in RB19 dye removal efficiency. Therefore, it can be concluded that the biochar @ZnO/Ce photocatalyst can be promisingly applied for the removal of azo dyes in aqueous solutions.

## Introduction

Recently, surfactants, dyes and heavy metals have been broadly employed and, accordingly, their input into the ecology poses many hazards to plants, human health, and the climate^1^. Significantly, wastewater containing synthetic dyes endangers the health of the ecosystem, humans and aquatic animals. These substances can affect the light activity of aquatic plants while reducing light penetration, causing eutrophication, increasing suspended solids, and increasing turbidity in water bodies^2,3^. Some dyes also degrade in wastewater under anaerobic conditions to form aromatic amines, which can be hazardous to human and animal health. The most significant issue with dyes lies in reactive and acid dyes^4^. Reactive dyes exhibit high solubility in water, and when used in 5–10% dyeing water solutions, they generate highly pigmented effluents that contribute to various environmental concerns. Moreover, these dyes have high chemical stability and very low biodegradability^5^. Hence, it is necessary to focus on efficiency and new approaches to eliminate these compounds from different types of wastewater^6^. There are several methods to remove reactive dyes from effluent. Presently, researchers are primarily concentrating on employing advanced oxidation methods for this purpose^7^. Among the various approaches within this technique, the photocatalytic oxidation method using nano-photocatalysts is very effective and has advantages in removing pollutants from industrial wastewater even at low concentrations^8^. In addition, photocatalytic process is an advanced oxidation procedure applied for photochemical degradation of toxic compounds and water purification^9^. In this sense, a heterogeneous photocatalyst is employed to destroy various families of hazardous substances^10^. The photocatalytic advanced oxidation process is an approach in which a strong oxidant such as hydrogen peroxide or ozone and a catalyst containing zinc oxide, iron, and manganese are used in presence or absence of an ultraviolet radiation source^11,12^. These procedures are based on the formation of free hydroxyl radicals with high oxidizing power, causing the conversion of organic chemical pollutants into inorganic substances^13,14^. When the energy of a photon is equal to or greater than the energy gap (E_g_) of the semiconductor, excitation of the electron from the valence band to the conduction band occurs, creating a hole in the valence band^15^. Excited electrons and holes can directly or indirectly produce hydroxyl radicals, which convert organic substances into inorganic substances^16,17^. This method is according to the generation of highly reactive kinds including hydroxyl radicals, which have high oxidation suitability with dye, causing dye degradation and removal from water and wastewater^18^. Photocatalysts are typically substances that lower the activation energy of a reaction, accelerating the cleavage of chemical bonds in organic compounds and, consequently, enhancing the efficiency of catalytic process^19,20^. It is worth mentioning that, hydroxide radicals are produced by chemical or photochemical reactions. Many photocatalysts—i.e. TiO_2_ and ZnO^21^ have been utilized to remove pollutants. The most important limitation of the efficiency of photocatalysts is adsorption and low surface area. Therefore, the decoration of semiconductors with appropriate foundations can overcome this limitation owing to the enhancement in charge segregation, rise in the lifetime of charge carriers, increase in surface charge transfer of adsorbent substrates, and cost reduction^22^.

Biochar is a substance attained from the thermal decomposition of biomass in an oxygen-free conditions^23^. Biomass comprises residual materials derived from diverse sustainable sources, such as plants, timber, agricultural crops, food waste etc. Owing to the porosity and the attendance of functional groups on the biomass texture, it can be turned into a material with bio-adsorbing properties, making it highly attractive as a substrate for photocatalytic oxidation^24^. On the other hand, biochar is a low-cost and green adsorbent^25^. Furthermore, this study encompasses nanotechnology, synthesis techniques, types of metals employed, the advantages of nanocatalysts, and prior research concerning the integration of nanocatalysts into biochar. Significantly, metal oxides can also be successfully loaded onto the primary biochar surface. Photocatalytic oxides including TiO_2_, ZnO, and Fe_2_O_3_ have been broadly employed to produce reactive oxygen species that are able to decompose water pollutant molecules^26,27^. Examinations have documented the application of oxides, including ZnO loaded on biochar, for the decomposition of various azo dye pollutants. When nanometer oxides are loaded into biochar, a composite with distinct hybrid properties is produced, exhibiting good performance for the photocatalytic reaction^28^. Because of the significant multiplicity of milkvetch plant kinds, it is employed to fabricate biochar in this study. Biochar can be incorporated with other materials, including ZnO/biochar^29^, biochar/TiO_2_^30^, biochar/BiOBr^31^, and MnFe-LDO–biochar^32^. Regarding doped ZnO nanoparticles, extensive investigations have been conducted^18,21^, according to which ZnO@Ce presents a widely employed catalyst used for structural, morphological, and photocatalytic applications^32,33^. However, as far as our knowledge extends, there has been no prior investigation into the utilization of ZnO@Ce in biochar for photocatalytic applications. Therefore, it becomes imperative to explore the experimental attributes of ZnO@Ce when incorporated into biochar as a photocatalyst. Hence, the synthesis and characterization of ZnO@Ce decorated on biochar for azo dye removal is the main innovation of the present study.

The principal objective of the current study is to prepare a novel and efficacious nanocatalyst for the removal of RB19. In this regard, the biochar@ZnO–Ce nanocatalyst was applied to remove RB19 dyes in the aquatic solution. As far as we know, the biochar@ZnO–Ce nanocatalyst was prepared and utilized for the first time in this scientific study. The physical attributes of the nanocomposite were explored using EDX, FTIR, TEM, XRD, FE-SEM, and BET procedures. In addition, the effects of key parameters such as pH, LED irradiance, H_2_O_2_ amount, nanocatalyst content, and initial RB19 amount were investigated. Moreover, the RB19 dye removal kinetics and adsorption isotherms were studies to determine the reaction kinetic and adsorption capacity of biochar@ZnO–Ce nanocatalyst. Furthermore, the reusability of the biochar@ZnO–Ce nanocatalyst was investigated in five runs.

## Materials and procedures

### Materials and chemicals

**Figure 1:**
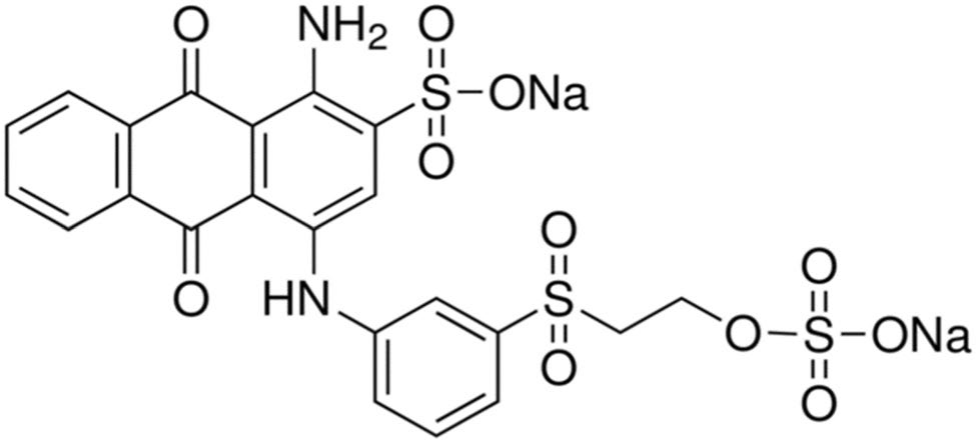
Chemical structure of RB19 dye.

The chemicals employed in this study include zinc nitrate hexahydrate (Zn(NO_3_)_2_⋅6H_2_O, > 99%), cerium nitrate hexahydrate (Ce(NO_3_)_3_⋅6H_2_O, > 99%),sodium carbonate (Na_2_CO_3,_ > 99%), sodium hydroxide (NaOH, > 98%), hydrochloric acid (HCl), and hydrogen peroxide (H_2_O_2_, 30%).It should be noted that all of the aforementioned chemicals were purchased from Merck Company. Deionized water was purchased and, then, used as solvent. Besides, Milkvetch plant (sourced from the forests found in Ilam, Iran) was gathered as the principal substance for the fabrication of biochar. Moreover, RB19 was synthesized by DyStar (Germany) as a contaminant to simulate synthetic effluent. Figure 1 illustrates the chemical structure of RB19.

### Preparation of RB19 solution

250 mg of dye was added to 500 mL of distilled water and, then, stirred for 1 h using a magnetic stirrer to prepare 500 ppm solution of RB19 dye. This solution was used to perform the experiments in different batches.

### Preparation of biochar

**Figure 2:**
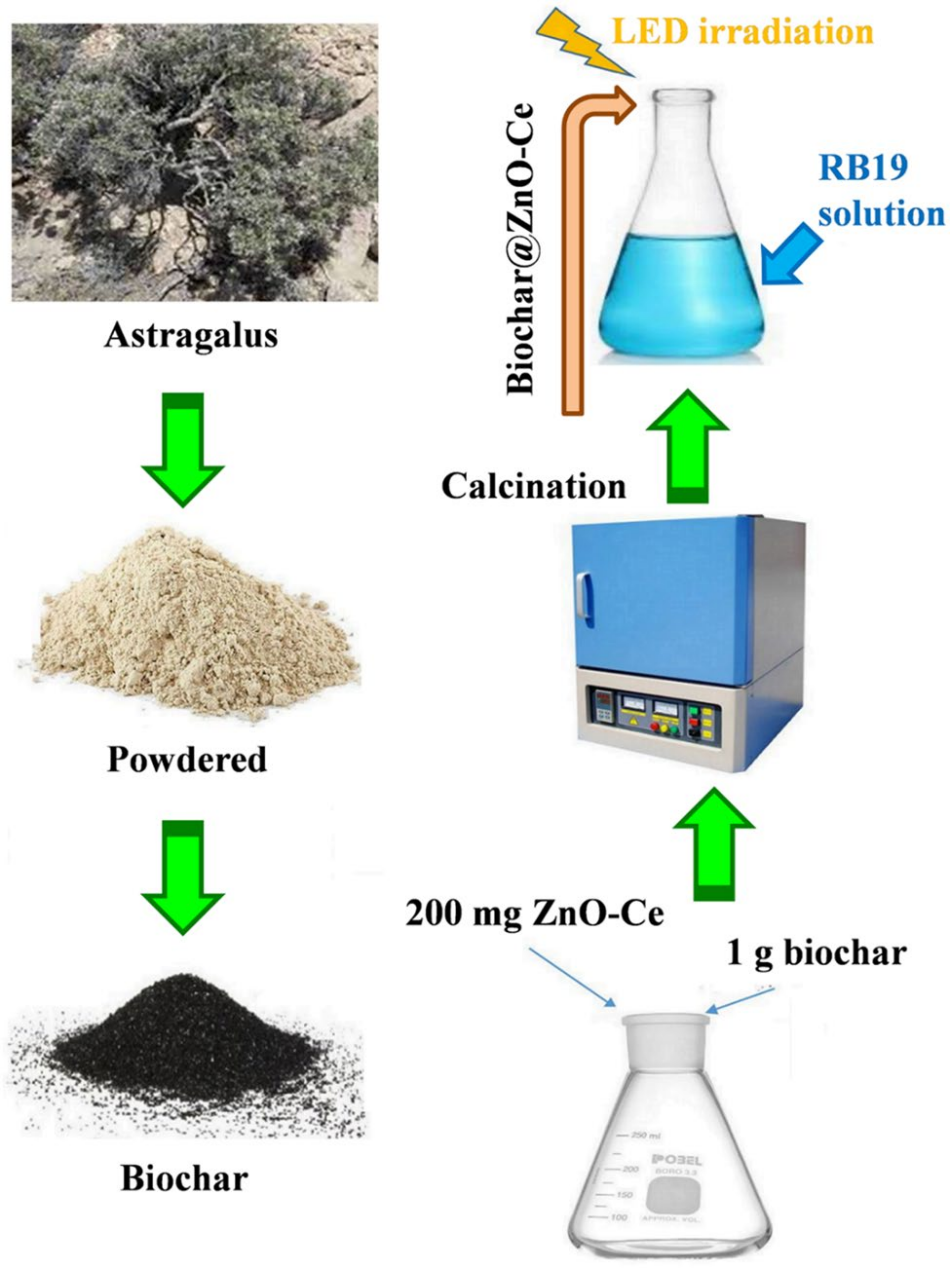
The preparation approach of the biochar@ZnO–Ce nanocatalyst and its utilization in RB19 dye removal.

Primarily, to remove impurities, the milkvetch plant was rinsed with deionized water and, then, parched in daylight for one week. Afterwards, it was fragmented into small pieces and subsequently subjected to heating in an oven at a temperature of 100 °C until a constant mass was achieved. Consequently, in order to activate the parched wood, it was sponged in H_3_PO_4_ (concentration of 95%) for 1 h. The ratio of dry wood to H_3_PO_4_ was determined to be 10:1. Furthermore, the activated precursor was put in a stainless-steel container and, then, heated at 650 °C for 1.5 h in a muffle furnace. Following that, the biochar was washed with distilled water until a pH level of 7 was reached and, then, the specimen was again positioned in the oven at 100 °C for 2 h. Finally, it was crushed and sieved and, then, particles between 50 and 100 mesh were applied to remove RB19 dye in the experiments. The schematic of the catalyst preparation approach and its utilization in RB19 dye elimination is displayed in Fig. 2.

### Synthesis of ZnO–Ce nanoparticles

Zinc-cerium oxide nanoparticles (Zn–Ce NPs) were synthesized while dissolving 14.9 g of zinc nitrate hexahydrate and 10.9 g of cerium nitrate hexahydrate in 100 mL of deionized water. Afterwards, 85 mL of Na_2_CO_3_was added drop by drop to the solution and continuously agitated at 70 °C for 2 h. Subsequently, filter deposits were rinsed multiple rounds with distilled water to extract pollutants. The nanoparticles were placed in an oven at 80 °C for 18 h to dry and remove water molecules. Ultimately, the specimen was calcined in a furnace at 500 °C for 3 h to convert zinc-cerium nitrate to Zn–Ce oxide and remove NOx gases and H_2_O, as well as enhance crystallinity.

### Synthesis of biochar@ZnO–Ce nanocatalyst

An amount of 1 g of biochar, in conjunction with 200 mg of ZnO–Ce nanoparticles, was introduced into a beaker containing 40 mL of deionized water. Then, the mixture was subjected to agitation for a duration of 2 h, employing a magnetic stirrer. Subsequently, the sample was placed to a porcelain plant and, then, dried in an oven at 100 °C for 12 h. Afterwards, the porcelain crucible containing the sample was calcined in a furnace at 250 °C for 3 h to synthesize zinc-cerium oxide nanoparticles decorated with biochar.

### Characteristics of nanocatalyst

In order to determine the textural and surface characteristics of biochar@ZnO–Ce, several approaches including SEM (Hitachi S-3400 N), EDX (VEGA II, TESCAN, Czech Republic), FTIR (Bruker, Vector 22), BET (Microtrac Bel Corp, Japan), and XRD (Bruker, D8 model) were carried out.

### The photocatalytic removal of RB19

A specific amount of the reactive blue 19 dye was dissolved in a beaker containing distilled water and, then, stirred on a magnetic stirrer. At first, the amount of dye at zero time was measured applying a spectrophotometer (Jasco-V630) at a wavelength of 600 nm. Subsequently, a precise amount of catalyst samples was introduced into the solution. The extent of color removal was then assessed by periodically extracting and examining the mixture at various time intervals, conducted in the absence of light. Afterwards, the sample underwent a photocatalytic procedure and was subsequently assessed under LED light. In this research, parameters affecting the reaction, including the concentration of reactive blue dye 19, the amount of biochar/ZnO–Ce catalyst, pH of the solution, the amount of LED lamp radiation, and the amount of H_2_O_2_ on the removal efficiency of RB19 dye were assessed. After performing each analysis, the removal percentage of RB19 dye was obtained using Eq. (1)^34^:

1 $$ {\text{RB19 }}\,{\text{removal (\% )}} = \frac{{C_{{0}} - C}}{C} \times 100 $$

where C_0_ and C are the initial concentration and final concentration of RB19 dye, respectively.

## Results and discussion

### Characteristics of the nanocatalyst

**Figure 3:**
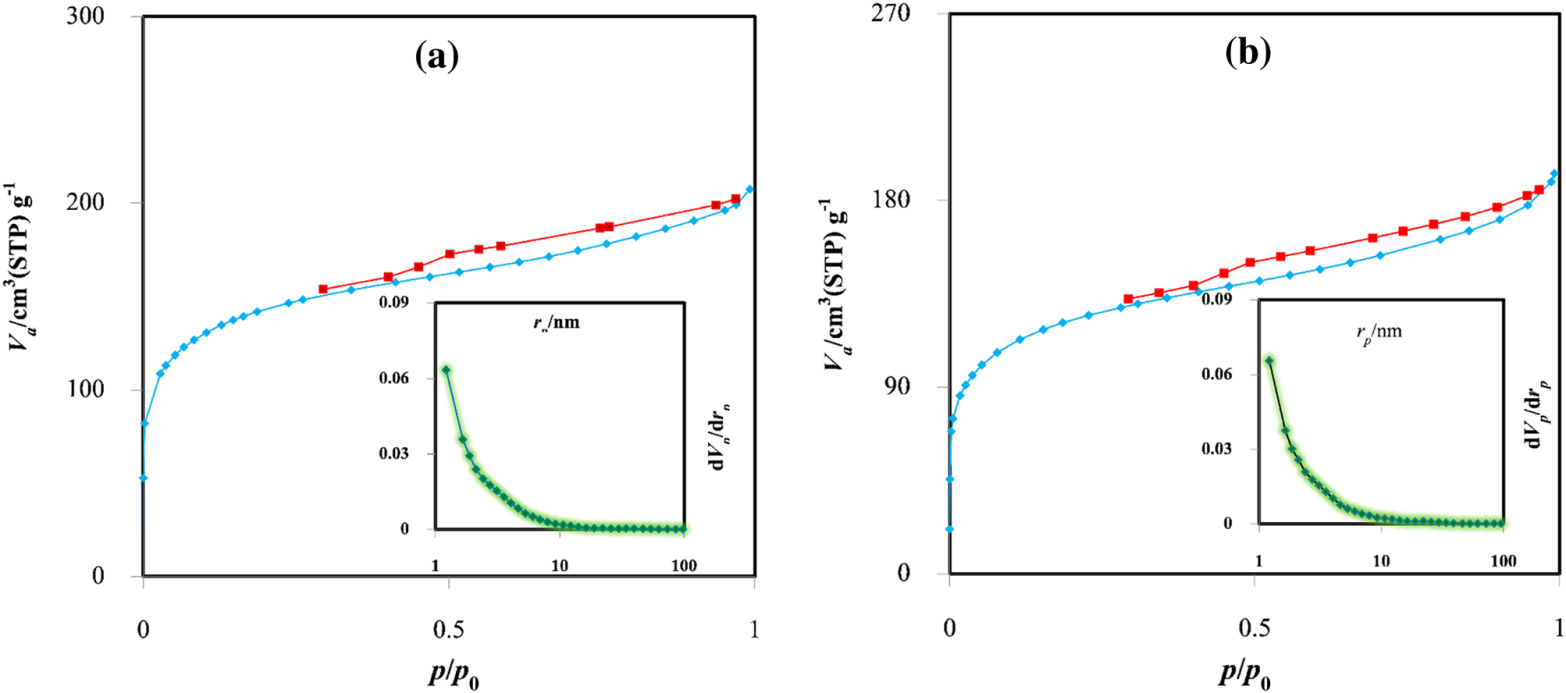
Nitrogen gas adsorption and desorption curve and pore size distribution (**a**) biochar, (**b**) biochar/ZnO–Ce nanocatalysts.

The catalytic activity is instantly proportionate to the essence of the porosity and its specific surface area. This characteristic enhances contact between the catalyst’s surface and the reactants. In general, utilizing biochar as a catalytic support will have a higher surface area as compared to mineral supports^35^. Figure 3 illustrates the adsorption and desorption isotherms of nitrogen gas for biochar and biochar/ZnO–Ce samples. As it is apparent from Fig. 3a,b, both Figures demonstrate adsorption isotherm of type (II) and exhibit hysteresis loops classified as type H3 based on IUPAC categorization. The type II isotherm illustrates the unlimited monolayer–multilayer adsorption^36^. The arrow point, the start of the nearly linear middle part of the isotherm, is often taken to demonstrate the step at which monolayer coverage is complete and multilayer absorption begins. Likewise, at very low pressures, micropore are filled with nitrogen gas. The turning point of the isotherm happens near the end of the first adsorbed monolayer. With the rise in relative pressure, additional layers, such as the second and subsequent ones, form until the maximum number of absorbed layers is reached in the saturation state^36,37^.

The characteristics of both biochar and the biochar decorated with ZnO–Ce oxide are presented in Table 1. As demonstrated in Table 1, S_BET_ for biochar and biochar@ZnO–Ce was 519.75 and 636.52 m^2^/g, respectively, which reveals that both substances have a remarkable specific surface area. Another important attribute of these substances is S_Langmuir_, which was 716.07 and 775.18 m^2^/g, confirming the high potential of these substances in eliminating pollutants^38^. Furthermore, the average pore diameter of biochar (2.48 nm) and biochar@ZnO–Ce (2.59 nm) shows that both materials have a mesoporous structure because their pore sizes are between 2 and 50 nm^9,37^. Moreover, the pore volume of biochar and biochar@ZnO–Ce was 0.331 and 0.405 cm^3^/g, respectively, which are very appropriate values for photocatalytic processes.

**Table 1:** BET outputs for biochar and biochar@ZnO–Ce.

Feature	Catalysts	
	Biochar	Biochar@ZnO–Ce
S_BET_ (m^2^/g)	519.75	636.52
Pore volume (cm^3^/g)	0.331	0.405
Mean pore diameter (nm)	2.48	2.59
S_Langmuir_ (m^2^/g)	716.21	775.18

**Figure 4:**
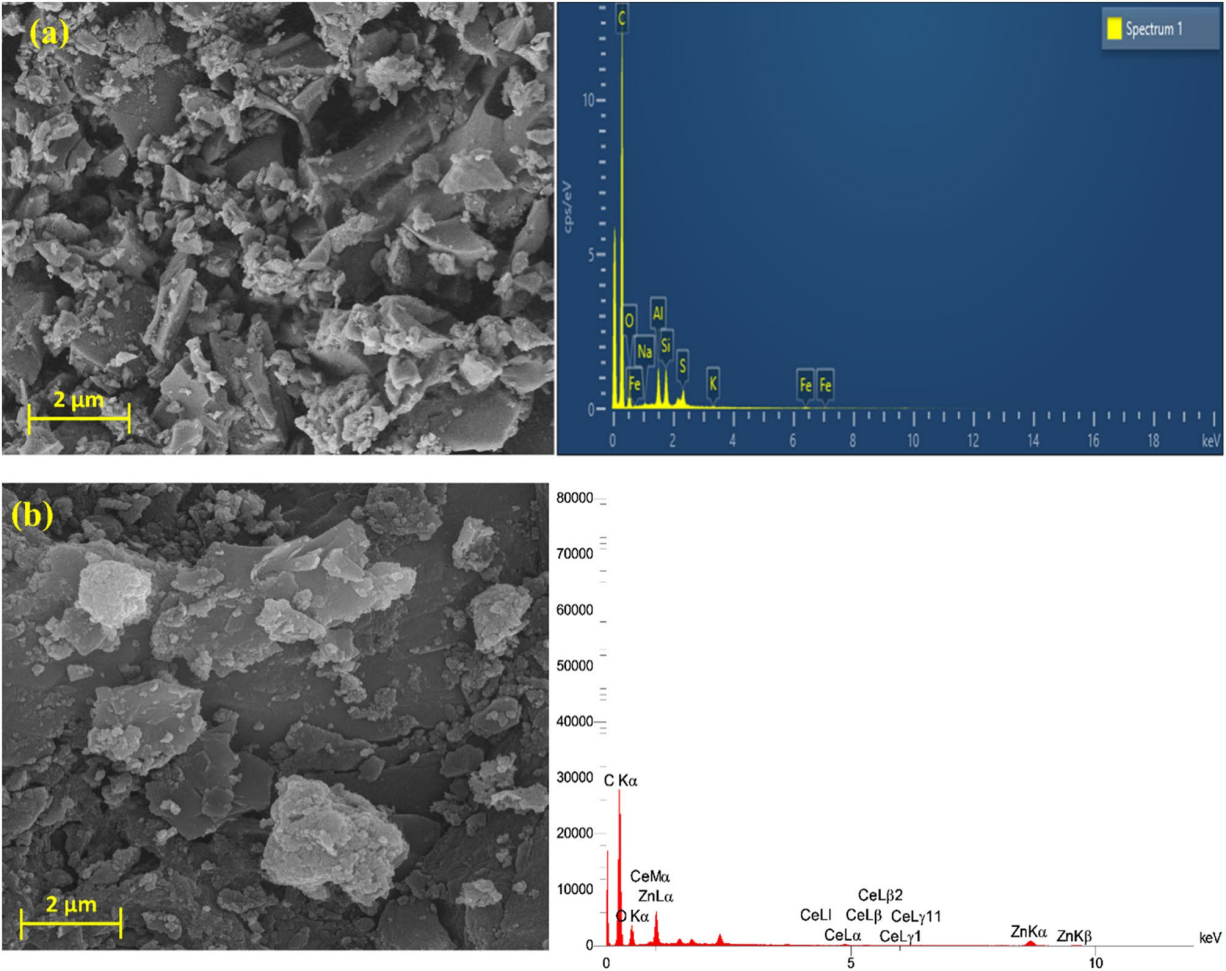
SEM and EDX images for (**a**) biochar, and (**b**) biochar@ZnO–Ce nanocatalyst.

SEM and EDX studies can be applied to estimate the morphology and surface distinctions of biochar and biochar@ZnO–Ce catalysts, as illustrated in Fig. 4. The numerical outputs of EDX are also demonstrated in Table 2. According to SEM image (a), a wide variety of cavities can be noticed on the surface of biochar, confirming that its surface is very porous. Moreover, there are many bumps, ups, and downs in the biochar texture, which demonstrates that the biochar support is very appropriates for eliminating the pollutants^38^. After the biochar surface was decorated with ZnO–Ce nanoparticles, its surface morphology was modified and, also, some cavities were covered with these metals. As illustrated in Fig. 4b, it can be clearly observed that the ZnO–Ce nanoparticles are correctly placed on the biochar. The SEM image in Fig. 4b reveals the structure of several irregular uneven layers including micropore and porous nature with grain boundaries formed on the surface of biochar. It is worth mentioning that ZnO–Ce nanoparticles had a spherical shape with a high degree of aggregation. The size of these agglomerated nanoparticles is in the nano range with an average diameter of 59 nm. In addition, EDX images were applied to identify various components in the texture of both biochar and biochar/ZnO–Ce samples, the results of which are demonstrated in Fig. 4a,b. Besides, the element percent in both catalysts is illustrated in Table 2, in which the weight percent of carbon and oxygen elements in biochar was 83.02 and 12.78%, respectively. The weight percent of other elements including Si, S, Fe, Al, Na, and K was negligible. After the synthesis of the biochar/ZnO–Ce nanocomposite, the weight percentages of C, O, Zn, and Ce were acquired as 62.36, 31.55, 5.56, and 0.53%, respectively. The presence of Zn and C elements in the catalyst structure plays a crucial role in removing RB19 dye molecules^39^. Furthermore, C, O, and N elements play a vital role in the absorption process.

**Table 2:** EDX results for biochar, and biochar@ZnO–Ce nanocatalysts.

Element	Biochar		Biochar@ZnO–Ce	
	W (%)	A (%)	W (%)	A (%)
O	12.78	10.18	31.55	27.19
C	83.02	88.02	62.36	71.59
Zn	0	0	5.56	1.17
Ce	0	0	0.53	0.05
Al	1.38	0.65	0	0
Si	1.42	0.64	0	0
Na	0.11	0.06	0	0
Fe	0.34	0.08	0	0
K	0.07	0.02	0	0
S	0.87	0.34	0	0
Total	100	100	100	100

*W* weight percent, *A* atomic percent.

**Figure 5:**
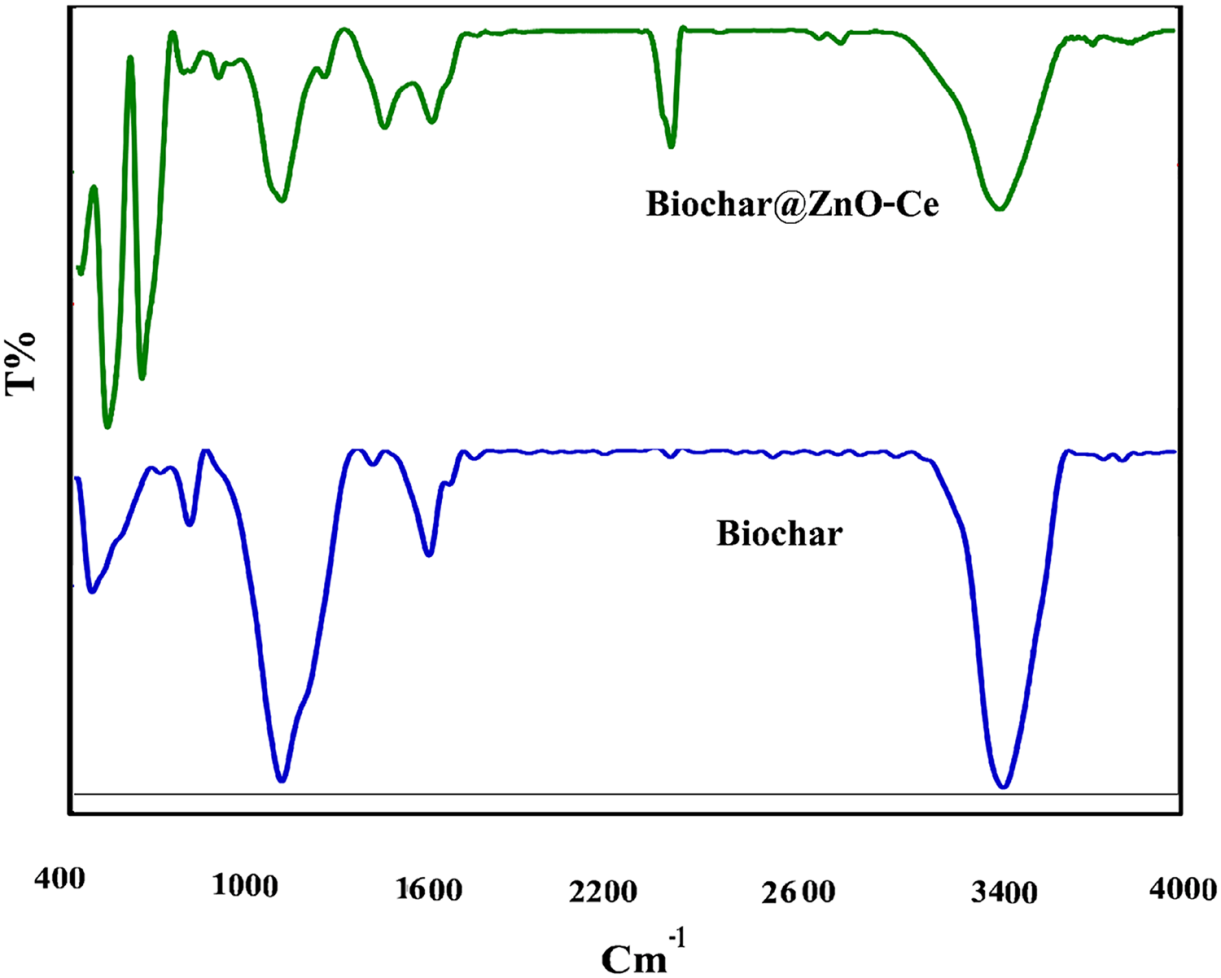
FTIR spectrum of biochar and biochar@ZnO–Ce nanocatalyst.

According to Fig. 5, the FTIR spectrum was utilized to specify functional groups in biochar and biochar@ZnO–Ce structures, respectively. Several peaks in biochar structure were observed at 469, 787, 1078, 1566, and 3441 cm^−1^, which are assigned to C=O, C–H, C–OH, O–H, and O–H functional groups, respectively^40^. These peaks were previously noticed in the configuration of the activated carbon^41^. The bands noticed at 3400 and 3500 cm^−1^ are due to stretching and deformation of the O–H hydroxyl bond due to water molecules absorbed on the surface of the sample^39^. In addition to these peaks, other peaks with low intensity were observed in the biochar@ZnO–Ce structure. As noticed, two diminutive peaks at 2357 and 2985 cm^−1^ were seen in Fig. 5, which can be attributed to the asymmetric vibration of C–H_2_ and CO_3_^2−^ vibration, respectively. Other peaks at 691, 1091, and 1567 cm^−1^ are assigned to C–H, C–OH, and O–H. The existence of these peaks is very important in the absorption procedure^42^.

**Figure 6:**
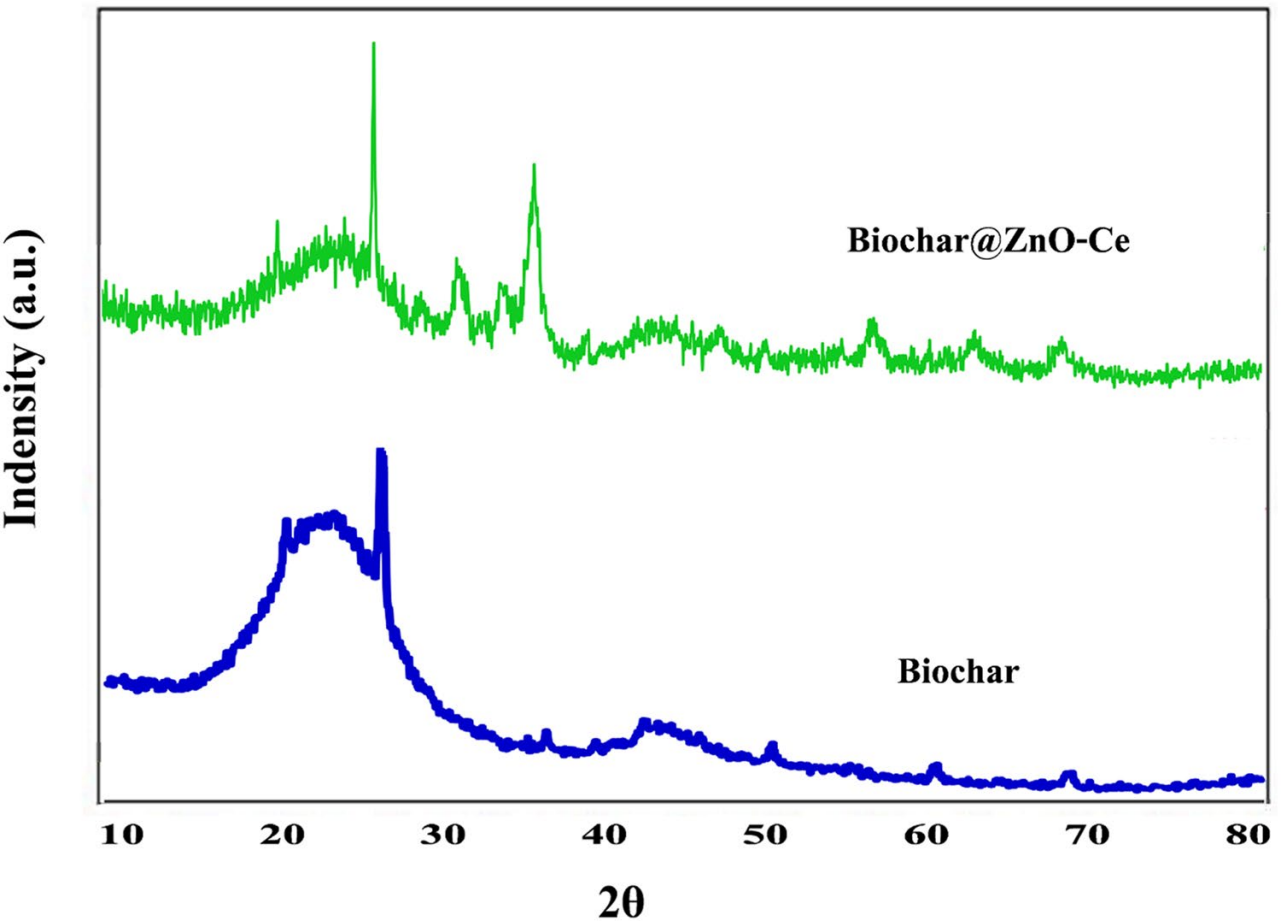
XRD pattern of biochar and biochar@ZnO–Ce nanocatalyst.

Furthermore, the outcomes of X-ray diffraction (XRD) pattern analysis for biochar and biochar@ZnO–Ce is exhibited in Fig. 6. As demonstrated in this Figure, some peaks in the biochar structure were observed at 21, 26, and 43°, which correspond to (002), (100), and (200) crystal planes. Moreover, tiny peaks were identified at 36.5, 50.4, 60.3 and 68°, which belong to (101), (102), (103) and (201), respectively. These peaks which can be attributed to JCPDS card number 036-1451^41,42^ demonstrate the high crystallinity of the biochar structure. In addition to the peaks related to biochar, the peaks observed at 7.31, 4.34, 5.47, 5.56, 8.62, and 68.3° are related to ZnO, which can clearly prove the attendance of ZnO on biochar. Additionally, these peaks correspond to JCPDS card numbers 04-0873. Likewise, a new peak at θ = 29.2° can be seen in the XRD patterns of the biochar@ZnO–Ce sample, confirming Ce_2_O_3_/CeO_2_ (crystal plane 111) as a new phase. Most Ce ions cannot integrate into the ZnO lattice owing to the larger ionic radius of Ce^3+^ as compared to Zn^2+^. Thus, Ce ions are dispersed in the form of Ce_2_O_3_ and CeO_2_ on the surface of biochar and zinc oxide. The average crystal size calculated for the synthesized ZnO–Ce nanoparticles was 24.13 nm. Comparing the structures of biochar and biochar@ZnO–Ce shows that biochar@ZnO–Ce has a more crystalline structure than biochar due to the fact that there are more substantial peaks in the structure of biochar@ZnO–Ce, indicating that its texture is highly crystalline^43^.

**Figure 7:**
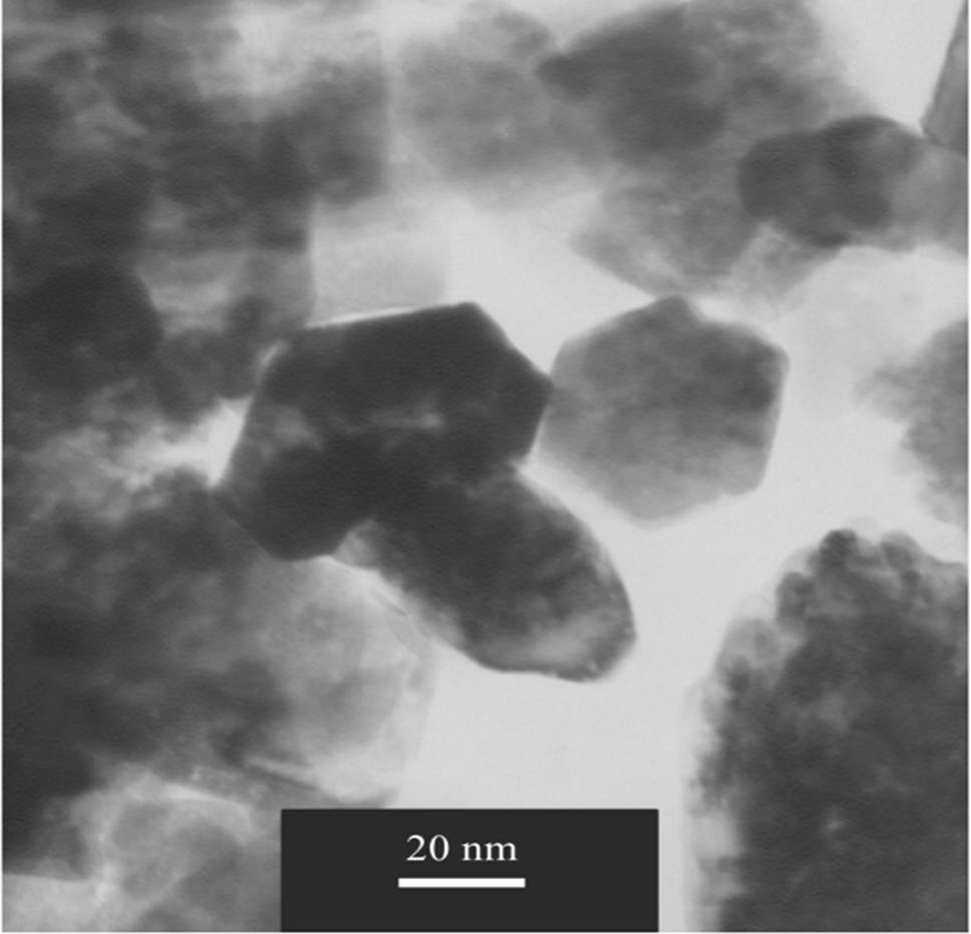
TEM image for biochar@ZnO–Ce nanocatalyst.

Additionally, TEM analysis for biochar@ZnO–Ce photocatalyst is demonstrated in Fig. 7, which indicates that the particle size is lower than 20 nm. Moreover, this figure reveals that particles have hexagonal and comparable forms. Hence, the biochar@ZnO–Ce nanocatalyst was nanoscale.

### Analysis of the absorption spectrum of biochar@ZnO–Ce nanocatalyst

**Figure 8:**
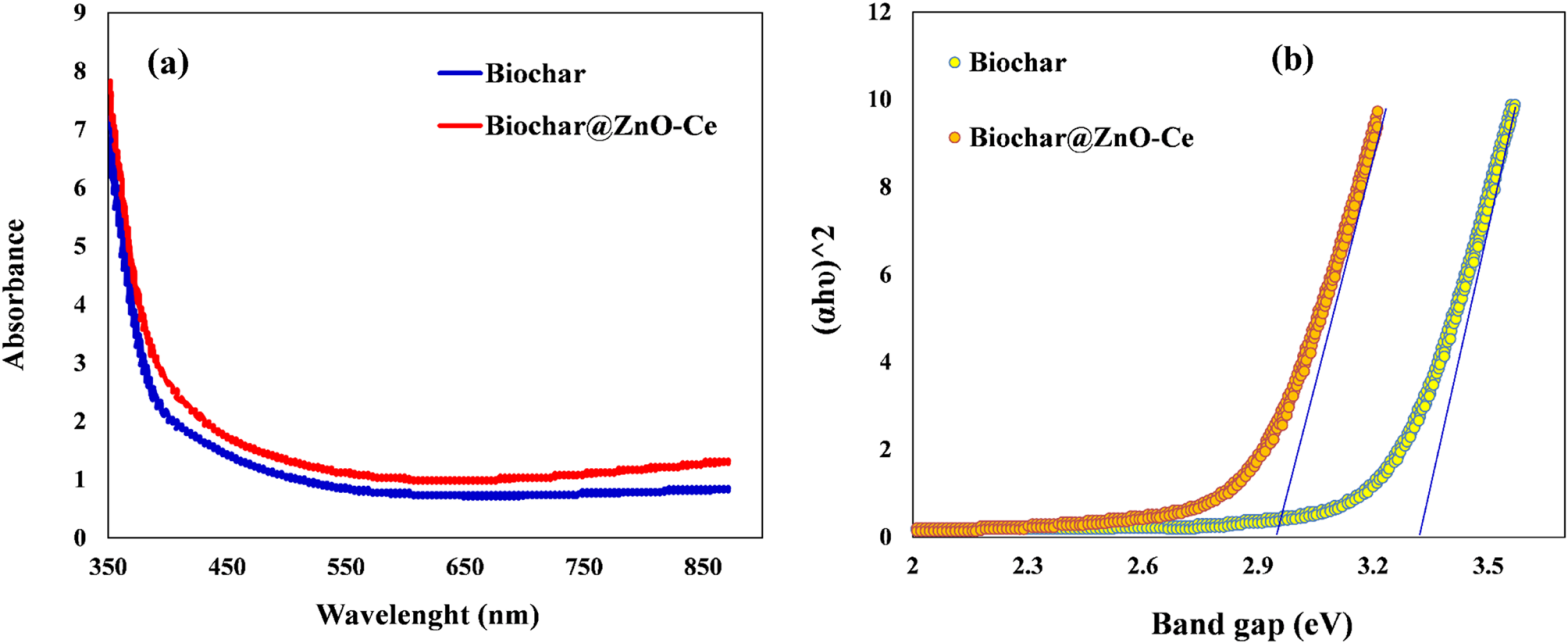
(**a**) UV–Vis absorption spectrum and (**b**) band gap of biochar and biochar@ZnO–Ce nanocatalyst.

The absorption spectrum of biochar and biochar@ZnO–Ce nanocatalyst is displayed in Fig. 8a, which shows that the absorption spectrum of biochar changes is the result of ZnO–Ce incorporation into the biochar@ZnO–Ce nanocatalyst. The band gap of photocatalysts can be specified using UV–Vis absorption spectrum and Eq. (2)^44^:

2 $${\left(\alpha h\vartheta \right)}^{2}=A\left(h\vartheta -{E}_{g}\right),$$

where α is the absorption coefficient (cm^−1^), $h\vartheta$ is the photon energy (eV), A is the constant of the equation, and E_g_ is the energy of the band gap^43,44^. Through irradiating LED on the biochar@ZnO–Ce nanocatalyst, photons with an energy similar to or more than the band gap are absorbed, which results in inducing the stimulation of electrons from the valence band to the conduction band. Hence, by drawing (αℎ*v*)^2^ versus ℎ*v* and with the assistance of linear extrapolation, band gap quantities are attained. It can be observed in Fig. 8b that regarding the incorporation of ZnO–Ce nanoparticles into biochar the band gap of the composite has dwindled from 3.31 to 2.96 eV. ZnO–Ce, as a sub-balance, which causes a distinct energy level between the two resonance and valence bands, leads to a decline in the band gap of biochar@ZnO–Ce nanocatalyst. Significantly, it confirms that, by diminishing the band gap, the biochar@ZnO–Ce shows higher photocatalytic activity than biochar^45^.

### Photocatalytic performance of biochar@ZnO-Ce nanocatalyst

#### The impact of initial concentration of RB19

**Figure 9:**
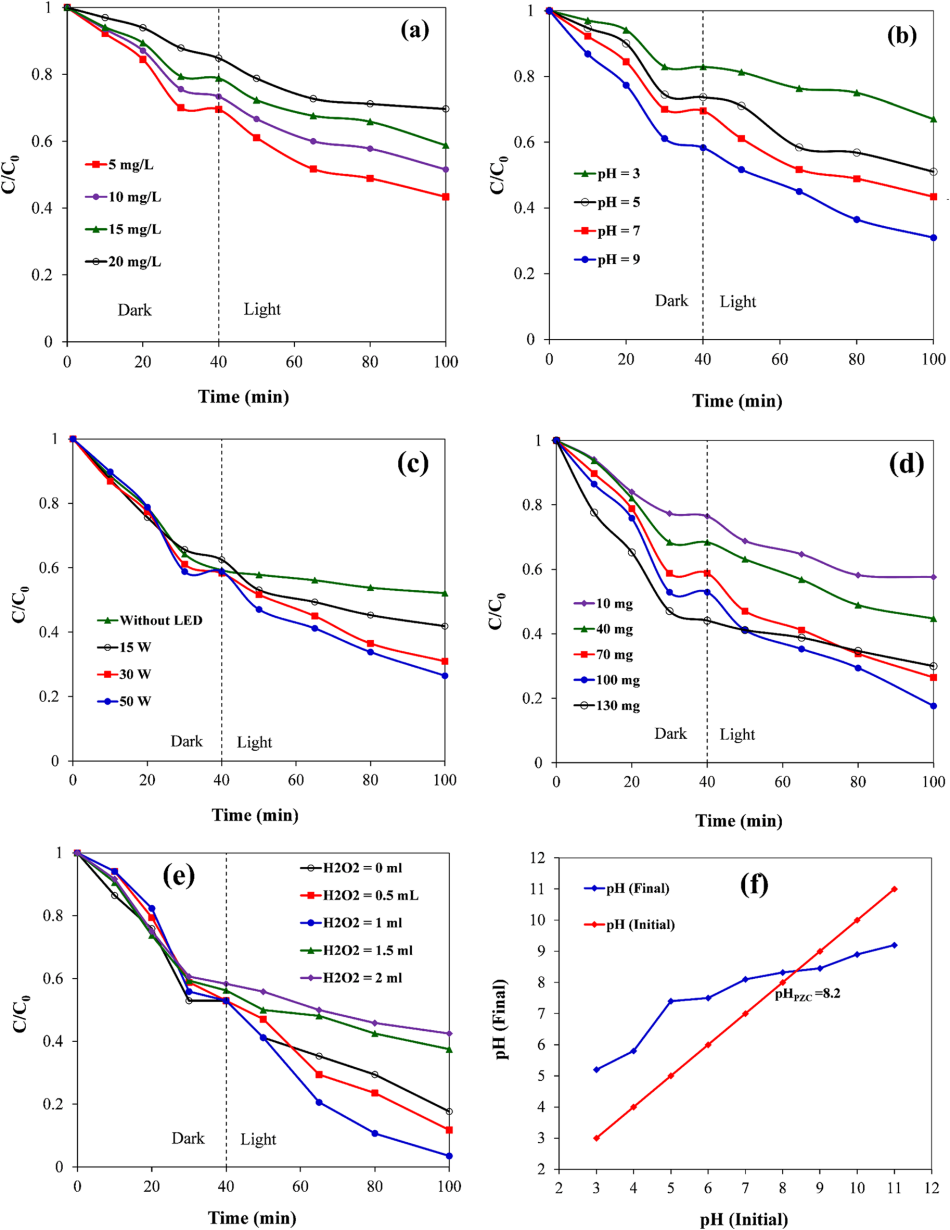
(**a**) impact of initial dye concentration, (**b**) impact of pH, (**c**) impact of light source power (**d**) impact of photocatalyst content, (**e**) impact of H_2_O_2_ dose on the elimination rate of RB19 dye, and (**f**) pH graph at the isoelectric point (in pH 9, concentration: 5 ppm, photocatalyst dose: 70 mg, solution volume: 100 mL).

Figure 9a demonstrates the amount of C/C_0_ (removal rate) in both dark and light conditions in terms of time and in different initial dye concentrations. As can be seen, C/C_0_ values decrease with time. The rate of photocatalytic decomposition of dyes depends on the possibility of OH* radical formation on the surface of biochar/ZnO–Ce nanocatalyst and the reaction of dye molecules with ^⋅^OH radical. At the beginning of the reaction, while increasing in the initial concentration of the dye, the degradation rate increases, which may be due to the increment in the possibility of the reaction between the dye molecules and the ^⋅^OH radical^44^. However, a further increase in the concentration of the dye leads to a decline in the activity of the catalyst, which is the reason for this phenomenon, the inhibition of the reaction between the dye molecules and the ^⋅^OH radical^45^. Therefore, at high dye concentrations, more dye molecules are absorbed on the catalyst’s surface, which leads to the reduction of ^⋅^OH radical formation in the reaction medium^46^.

#### Impact of pH

Considering the photocatalytic reaction of dye removal, pH is one of the most significant variables that can affect the speed of the dye removal procedure in different ways. Since the surface charge of the photocatalyst is dependent on the pH of the solution, the pH can have a remarkable impact on the dye adsorption on the photocatalyst surface^47,48^. The analysis examines the effects of pH on the yield of the photocatalytic degradation procedure, considering three reaction mechanisms that contribute to dye decolorization, i.e. (1) hydroxyl radical assault, (2) direct oxidation by the positive hole, and (3) direct reduction by the electron in band conductive. Additionally, in order to investigate the effect of pH on the color removal yield, it was investigated using HCL and NaOH solutions as well as pH in the range of 3, 5, 7 and 9, as depicted in Fig. 9b. As can be noticed, at pH 9, the slope of the graph is higher, which reveals that at this pH, more dye removal is achieved. Therefore, it can be expressed that the optimal pH is pH 9. Below the isoelectric point, the photocatalyst surface carries a positive charge, while above this point, it bears a negative charge^48,49^. To determine pH_PZC_, the pH_final_ graph should be drawn in terms of pH_initial_ (Fig. 9). The intersection of these two graphs is the pH _PZC_ or pH point of zero charge. The surface charge of the photocatalyst is determined via PZC, which is defined as pH_PZC_. At this pH, the positive charges on the surface of the photocatalyst are equal to the negative charges^50^.

**Figure 10:**
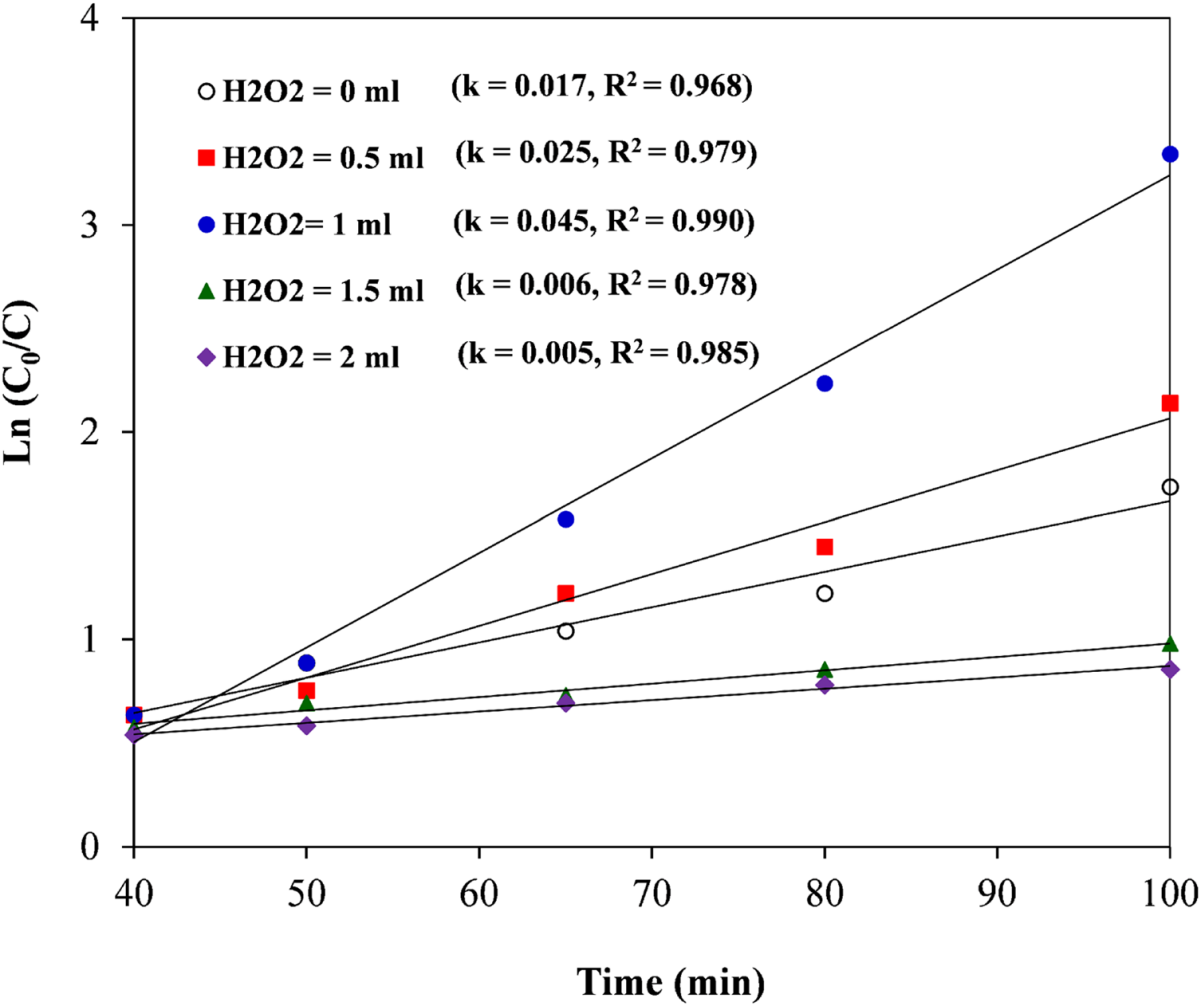
Kinetic study of RB19 removal utilizing biochar@ZnO–Ce nanocatalyst.

As can be seen in Fig. 10, pH_PZC_ is equal to 8.2, which means that, below this value, the surface of the photocatalyst has a positive charge and above this point, it has a negative charge. Accordingly, in pH_PZC_ > pH, dye pollutants with a negative charge are adsorbed because the surface of the photocatalyst has a positive charge. Considering that pH 9 was calculated as the optimum pH in this work, it can be demonstrated that RB19 has a positive charge (cationic dye) in an aqueous solution.

#### Impact of LED power

Figure 9c represents the impact of LED irradiation power on the removal yield of RB19 dye. This figure demonstrates that the amount of dye removal increases with promoting power. Therefore, an LED lamp with a power of 50 W was used to remove 65% of the RB19 dye, while lamps with powers of 30 and 15 W removed only 30 and 20% of the RB19 dye, respectively. The reason for this state is that by boosting the LED irradiation power and (as a consequence) the severity of light ameliorates, the particles of the biochar@ZnO–Ce nanocatalyst become eager to produce more ^⋅^OH radicals which results in an improvement in the efficiency of the photocatalytic reaction^46,48^.

#### Effect of nanocatalyst concentration

Photocatalytic removal of RB19 in an aqueous mixture was executed utilizing various concentrations of biochar@ZnO–Ce nanocatalyst (30, 45, 60 and 75 mg), as depicted in Fig. 9d. From this figure, it can be seen that photocatalytic removal efficiency also rises with the boost of the amount of nanocatalyst. The main reason for this is the availability of active sites of the biochar@ZnO–Ce nanocatalyst for the progress of the photocatalytic procedure. Increasing the nanocatalyst content to 100 mg yields the highest dye removal rate. However, with a further increase in the adsorbent amount (to 130 mg), the dye removal efficiency diminishes. This is due to the accumulation of organic mediators in the pore and on the surface of the photocatalyst, which affects the absorption of dyes and reduces the photocatalytic activity of the nanocatalyst in doses higher than 100 mg^48,50^.

#### Impact of H_2_O_2_ amount

The impact of H_2_O_2_ dose (0, 0.5, 1, 1.5, and 2 mL) on the removal of RB19 dye was surveyed. In this regard, other variables including biochar@ZnO–Ce content of 100 mg, RB19 content of 5 mg/L, LED power of 50 W, and pH of 9 were kept constant. Figure 9e reveals the influence of H_2_O_2_ dose on RB19 dye elimination yield. As revealed, the removal yield of RB19 dye rises while boosting the dosage of H_2_O_2_ and, accordingly, the optimum dose of H_2_O_2_ was acquired as 1 mL. Owing to the appropriate generation of hydroxyl radicals and the recombination inhibition of electrons and holes (e^−^/h^+^) that appears during the photocatalytic process, H_2_O_2_ rises the removal efficiency of RB19^44,49^. The mechanism can be observed in Eqs. (3)–(5). In the mentioned equations, *hv* and ${e}_{CB}^{-}$ are the photon energy and conduction band electrons, respectively^50,51^.

3 $${\mathrm{H}}_{2}{\mathrm{O}}_{2}+ {\mathrm{O}}_{2}^{.-} \to {\cdot}^\mathrm{OH}+ {\mathrm{OH}}^{-}+ {\mathrm{O}}_{2},$$

4 $${\mathrm{H}}_{2}{\mathrm{O}}_{2}+\mathrm{hv }\to {2}^{.}\mathrm{OH},$$

5 $${\mathrm{H}}_{2}{\mathrm{O}}_{2}+{e}_{CB}^{-}\to {\cdot}^\mathrm{OH}+ {\mathrm{OH}}^{-}.$$

### Kinetic study

Generally, equilibrium is not immediately established in porous absorbents. Accordingly, the mass transfer from the solution to the active sites of the adsorbent particles is limited via the mass transfer resistances. This indicates that it takes time to reach equilibrium. The progress of time in the absorption process is known as absorption kinetics. Hence, research on adsorption kinetics is necessary to characterize the rate-limiting mass transfer mechanism and to assess the mass transfer parameters^52,53^. The RB19 removal procedure utilizing the biochar@ZnO–Ce nanocatalyst was scrutinized under two various circumstances: LED lights for 60 min and in the dark for 40 min. In the absence of an LED lamp, the surface absorption procedure was prevailing, while under LED light, the photocatalytic approach played a substantial role. To investigate the kinetic characteristic of RB19 dye elimination via biochar@ZnO–Ce nanocatalyst, the RB19 dye concentration was evaluated at diverse time intervals. As demonstrated in Fig. 10, the kinetics of the photocatalytic process follow the Langmuir–Hinshelwood (L–H) procedure. The L–H model suggests a pseudo-first order kinetic equation (Eq. 6) for such nanocatalytic reactions^54^.

6 $$ {\text{ln}}\,\left( {\frac{{C_{0} }}{{C_{t} }}} \right) = k_{app} \,t, $$

where C_0_ and C_t_ are the RB19 content (mg/L) at times 0 and t, respectively, *k* and *t* are the apparent rate constant (min^−1^) and reaction time (min), respectively. Figure 10 exhibits the linear relationship between the RB19 content and the LED irradiation time in presence of H_2_O_2_. The slope represents the rate constant of the reaction (*k*) and is a criterion of photocatalytic activity^55^. According to Fig. 10, it can be seen that the highest reaction rate constant (*k* = 0.045 min^−1^) and correlation coefficient (R^2^ = 0.99) are achieved in 1 mL of H_2_O_2_. Radical species produced during semiconductor optical excitation are accountable for the removal of dyes. The principal stages can be revealed in the following steps (7–13)^55,56^:

7 $$ {\text{Conductive }} + {\text{Photons }}\left( {{\text{hv}}} \right) \, \to {\text{e}}^{ - } \left( {{\text{CB}}} \right) \, + {\text{ h}}^{ + } \left( {{\text{VB}}} \right), $$

8 $$ {\text{h}}^{ + } + {\text{ H}}_{{2}} {\text{O }} \to {\text{ H}}^{ + } + {\text{ OH}}^{ - } , $$

9 $$ {\text{h}}^{ + } + {\text{ OH}}^{ - } \to {\text{ OH}}^{.} , $$

10 $$ {\text{e}}^{ - } + {\text{ O}}_{{2}} \to {\text{ O}}_{{2}}^{.} , $$

11 $$ {\text{2e}}^{ - } + {\text{ O}}_{{2}} + {\text{ H}}_{{2}} {\text{O}} \to {\text{ H}}_{{2}} {\text{O}}_{{2}} , $$

12 $$ {\text{e}}^{ - } + {\text{ H}}_{{2}} {\text{O}}_{{2}} \to {\text{ OH}}^{ - } + {\text{ OH}}^{.} , $$

13 $$ {\text{Organic pollution}} + {\text{OH}}^{.} + {\text{ O}}_{{2}} \to {\text{ CO}}_{{2}} + {\text{ H}}_{{2}} {\text{O }} + {\text{Other degradation products}}. $$

### Adsorption isotherms

**Figure 11:**
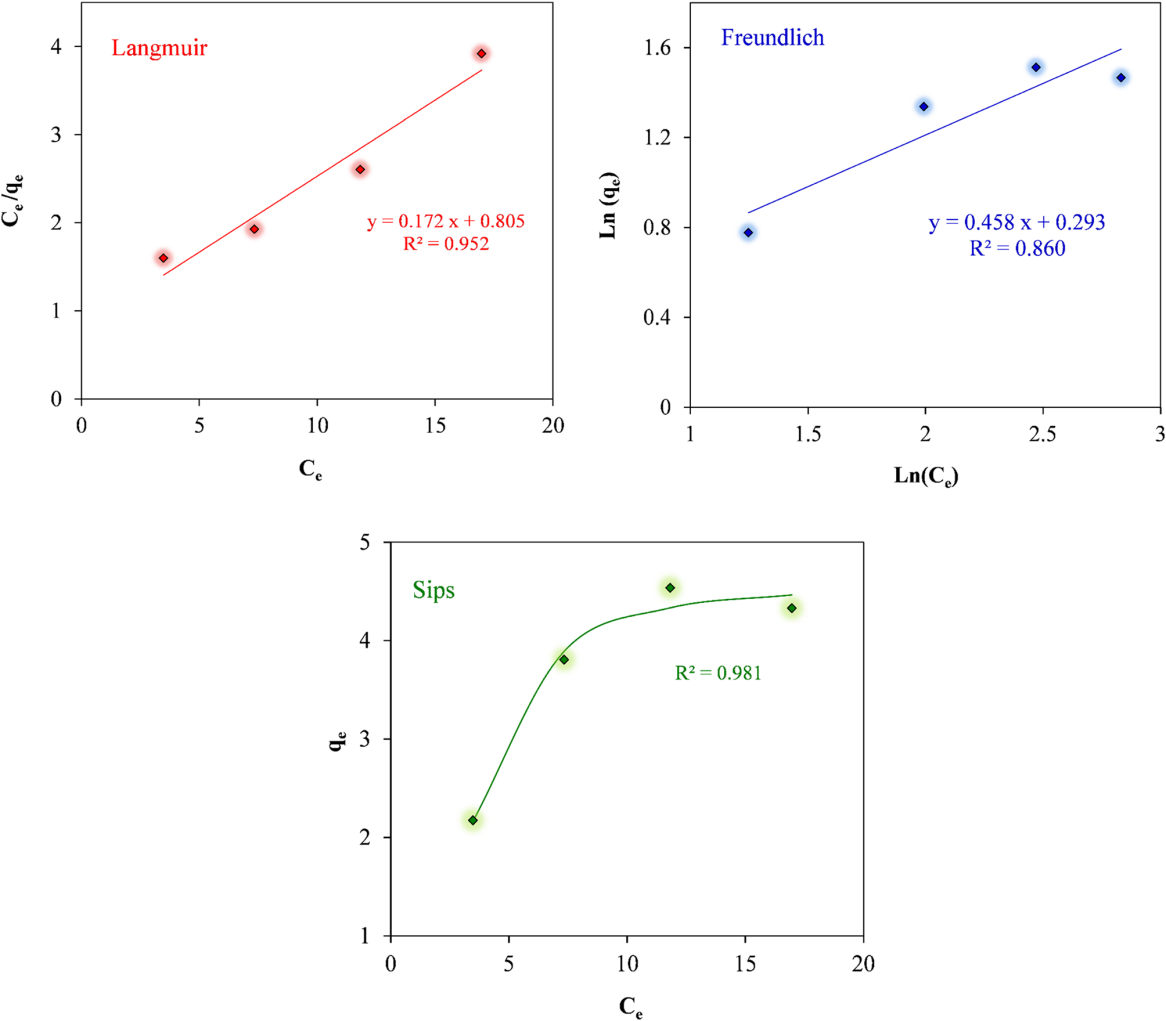
Langmuir, Freundlich, and Sips adsorption isotherms for RB19 removal utilizing biochar @ZnO–Ce nanocatalyst.

In order to comprehend the mechanism of RB19 dye adsorption on biochar/ZnO–Ce photocatalyst, Langmuir, Freundlich and Sips isotherms were employed to describe the relationship between the equilibrium adsorbed amount and its equilibrium concentration in the solution at a constant pH value. Langmuir, Freundlich and Sips isotherm forms presented via Eqs. (14), (15) and (16) respectively, were utilized to fit the adsorption equilibrium isotherm of RB19 dye onto biochar@ZnO–Ce nanocatalyst at room temperature as illustrated in Fig. 11 and Table 3. It is well-reported that Langmuir model considers monolayer adsorption on biochar@ZnO–Ce nanocatalyst with a homogeneous scattering of sorption pores, while the Freundlich model can be employed to better describe non-homogeneous surface adsorption^56^.

**Table 3:** Isotherm parameters of RB19 removal via biochar@ZnO–Ce nanocatalyst.

Langmuir isotherm				Freundlich isotherm				Sips isotherm			
q_m_ (mg/g)	K_L_ (L/mg)	R_L_	R^2^	K_f_ (mg/g) (L/mg)^1/n^	n	1/n	R^2^	q_m_ (mg/g)	K_s_ (L/mg)^n^	n	R^2^
5.8139	0.138	0.591	0.952	1.34	2.183	0.458	0.86	4.559	0.04	2.496	0.981

14 $$\frac{{C}_{e}}{{q}_{e}}=\frac{1}{{q}_{max}{K}_{L}}+\frac{1}{{q}_{max}}{C}_{e},$$

15 $$\mathrm{log}\left({\mathrm{q}}_{\mathrm{e}}\right)=\mathrm{log}\left({\mathrm{K}}_{\mathrm{F}}\right)+\frac{1}{\mathrm{n}}\mathrm{log}\left({\mathrm{C}}_{\mathrm{e}}\right),$$

where q_e_ is the number of RB19 dye molecules adsorbed on biochar@ZnO–Ce nanocatalyst at equilibrium. Q_max_ and *K*_L_ are the Langmuir constants for adsorption capacity and adsorption rate, respectively. K_F_ is the adsorption capacity of the adsorbent and n denotes the desirability of the adsorption procedure. The equation of Sips isotherm is also expressed as follows^57^:

16 $${q}_{e}=\frac{{\mathrm{q}}_{\mathrm{m}}{\mathrm{K}}_{\mathrm{s}}{\mathrm{C}}_{\mathrm{e}}^{\mathrm{n}}}{1+{\mathrm{K}}_{\mathrm{s}}{\mathrm{C}}_{\mathrm{e}}^{\mathrm{n}}}.$$

According to the comparison of R^2^ of all three models, it can be confirmed that the equilibrium amounts are more consistent with the Sips isotherm since this isotherm has the highest correlation coefficient (0.981). On the other hand, regarding the presented two-parameter models, it can be inferred that the Langmuir model is more appropriate and can satisfactorily express the absorption reaction of RB19^52,56^. Besides, the outcomes demonstrate that the maximum Langmuir adsorption capacity by biochar/ZnO–Ce nanocatalyst is 5.814 mg/g. In addition, the term n indicates that the adsorption of RB19 utilizing biochar/ZnO-Ce nanocatalyst is physical because its value is greater than 1. Moreover, the separation coefficient (R_L_) reveals that the absorption process of RB19 is favorable because its value is between 0 and 1^54,56^.

### Sensitivity analysis

**Figure 12:**
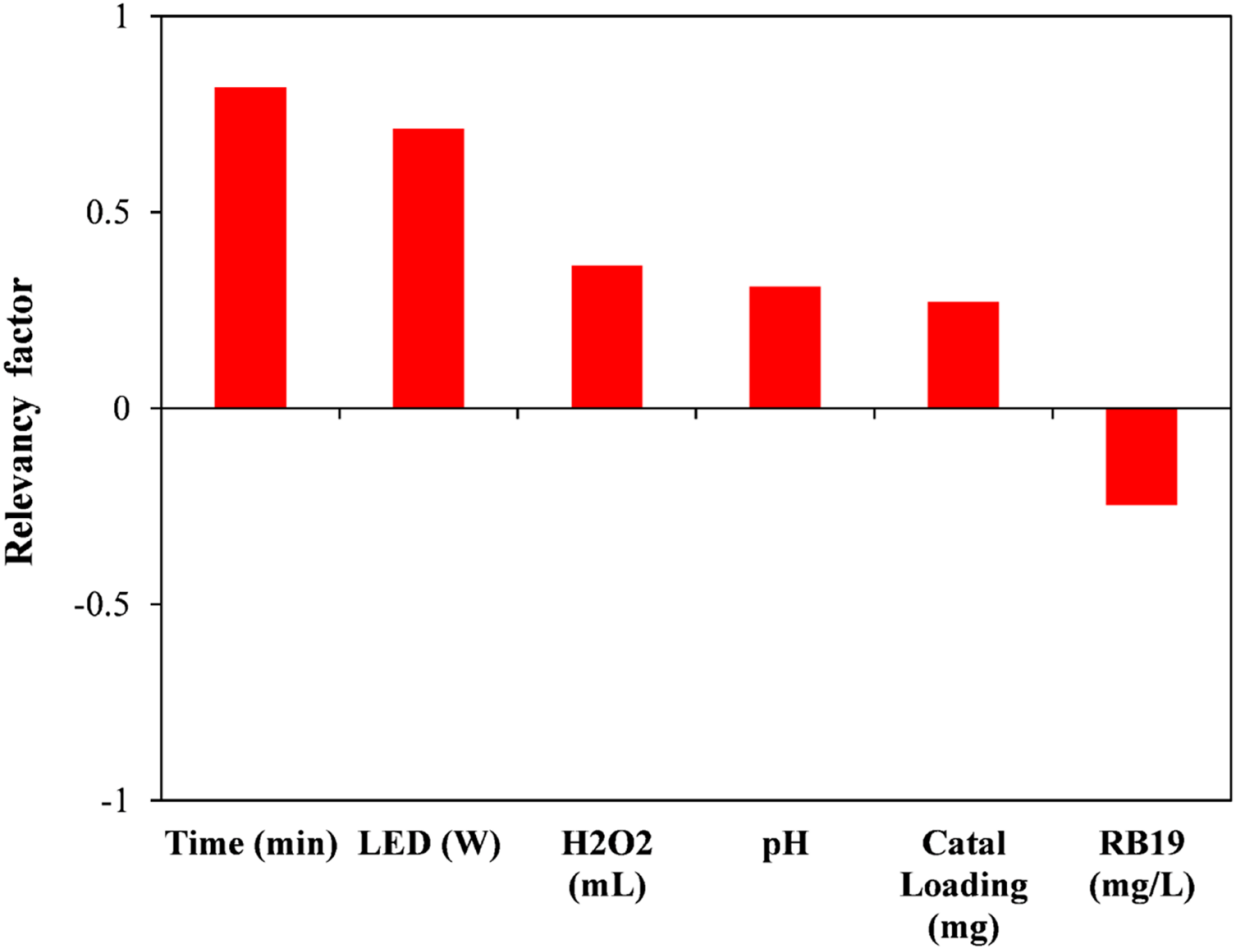
Pearson correlation coefficient between different input factors and RB19 dye removal percentage.

Examining the most significant variables affecting the efficiency of dye removal via the photocatalytic reaction can assist researchers in designing, simulating, and optimizing processes. Therefore, in this section, the experimental data measured for the removal percentage of RB19 dye have been employed to analyze the sensitivity of this parameter to different input factors. Accordingly, the Pearson correlation coefficient between each of the input factors and the color removal percentage was calculated, the results of which are exhibited in Fig. 12. This coefficient, which is a number between − 1 and + 1, reveals the degree of connection between two different factors^59^. It is worth mentioning that each of the two different parameters, X_1_ and X_2_, is a vector with n components. Accordingly, the Pearson correlation coefficient is calculated as follows (Eq. 17)^57^:

17 $$r\left({X}_{1},{X}_{2}\right)=\frac{\sum_{i=1}^{n}\left({X}_{1,i}-\overline{{X}_{1}}\right)\left({X}_{2,i}-\overline{{X}_{2}}\right)}{\sqrt{{\sum_{i=1}^{n}\left({X}_{1,i}-\overline{{X}_{1}}\right)}^{2}{\sum_{i=1}^{n}\left({X}_{2,i}-\overline{{X}_{2}}\right)}^{2}}}.$$

The positive or negative Pearson coefficient between the two variables indicates the direct and inverse relationship between the two factors, respectively. The values of + 1 and − 1 for this coefficient indicate the highest level of direct relationship and the highest level of inverse relationship, respectively^53,58^. According to Fig. 12, it can be observed that the dye removal percentage has a direct relationship with time, LED power, the addition of H_2_O_2_, pH, and photocatalyst dose, while it has an inverse relationship with the initial content of the dye. Furthermore, it is obvious from this figure that the percentage of RB19 dye elimination is more affected by the time and LED power.

### Comparison with other works

The comparison of the biochar@ZnO–Ce nanocatalyst activity with other nanoparticles in dye elimination is presented in Table 4. As revealed, the removal yield of RB19 utilizing biochar@ZnO–Ce is higher than most catalysts in prior investigations, demonstrating that the photocatalyst employed in this work has a remarkable performance to eliminate RB19. Besides, one of the benefits of the present study as compared to prior studies is the utilization of LED irradiation since others utilized more UV.

**Table 4:** Comparing the activity of biochar@ZnO–Ce with other nanoparticles in eliminating azo dyes.

Catalyst	Dye	Removal yield (%)	Reaction time	Ref.
CS-ZnS-NPs	Acid black 234	92.6	100 min	^48^
CS-ZnS-NPs	Acid brown	98	165 min	^48^
SiO_2_ NPs	Methylene blue	98	90 min	^49^
SiO_2_ NPs	Methyl orange	95	90 min	^49^
Biochar/Ag NPs	Methylene blue	96.09	140 min	^53^
ZnO/Al NPs	Rhodamine B	93.8	120 min	^54^
ZnO/Al NPs	Methylene blue	94.5	120 min	^54^
TiO_2_-GO NPs	Methylene blue	84	240 min	^55^
ZnAl_2_O_4_NPs	Congo red	98.3	40 min	^56^
CuFe_2_O_4_NPs	Methylene blue	93	100 min	^57^
ZnO@biochar	Methylene blue	95.19	20 min	^58^
Biochar@ZnO–Ce	Reactive blue 19	90.08	40 min	This study

### Recyclability of biochar@ZnO–Ce nanocatalyst

**Figure 13:**
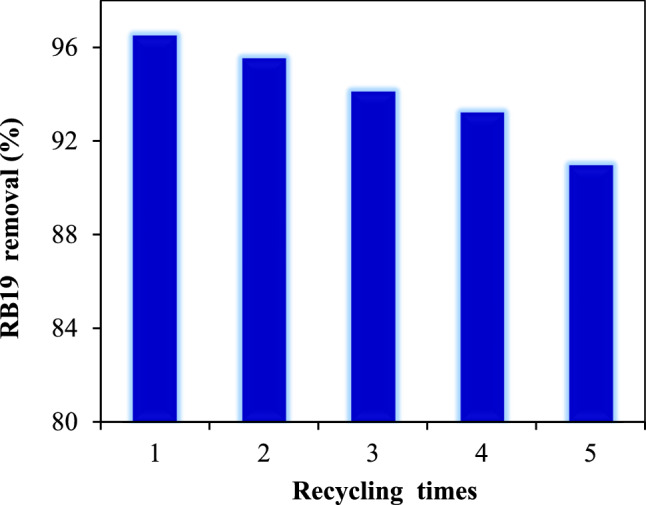
Recyclability of biochar@ZnO–Ce nanocatalyst in removal of RB19 from aqueous solution.

The recyclability of catalysts plays a vital role in their application in industrial scales^52^. The recyclability investigation for biochar@ZnO–Ce nanocatalyst was carried out in five reuse rounds, the results of which are demonstrated in Fig. 13. The analyses were conducted at RB19 content of 5 mg/L, pH 9, photocatalyst dosage of 100 mg, LED irradiation power of 50 W, and H_2_O_2_ dose of 1 mL. As indicated, the elimination percent of RB19 diminished by 90.39% (only a 6.08% reduction), demonstrating that the removal percentage has decreased by a negligible amount. Accordingly, the biochar@ZnO–Ce nanocatalyst has considerable recyclability and can be employed in considerable stages. Saraee et al. investigated the removal of methylene blue employing AC/Ag nanocatalyst in different concentrations of AC and Ag (1:2 and 1:3). After 5 reuse rounds, the removal efficiency of methylene blue utilizing AC/Ag nanocatalyst decreased from 94.7 to 88% (6.7% decline), demonstrating the low stability of this catalyst as compared to the present study^53,59^.

**Figure 14:**
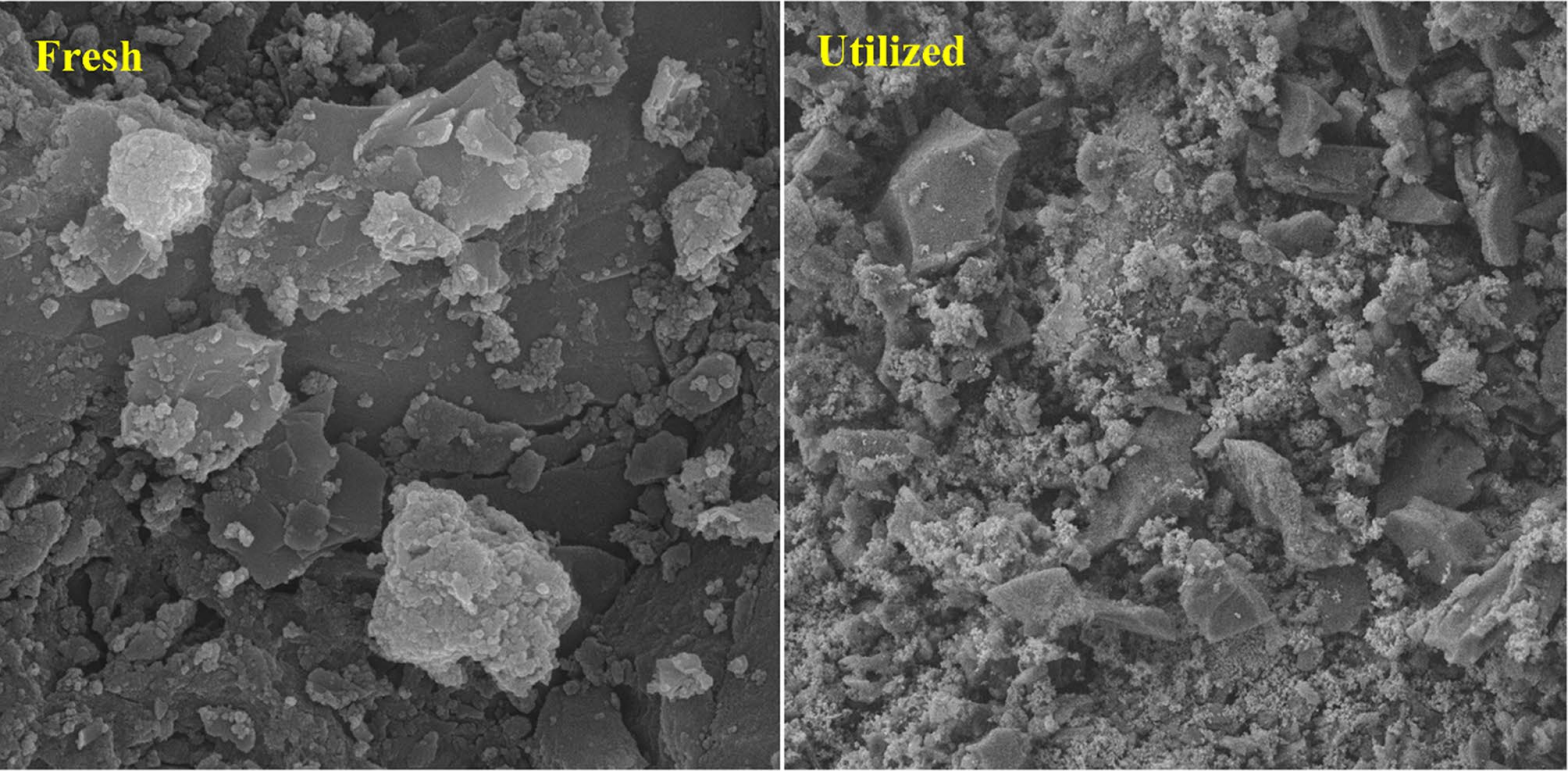
FE-SEM analysis fresh and after reuse of biochar@ZnO–Ce nanocatalysts (magnification equal to 2 µm).

After the recyclability examination, an FE-SEM test was conducted in order to identify the morphology and bumps on the biochar@ZnO–Ce nanocatalyst, the result of which is revealed in Fig. 14. Comparing the FE-SEM image (Fig. 14) before and after reuse, it can be noticed that the morphology of the biochar@ZnO–Ce nanocatalyst has changed after multiple cycles. Moreover, it is evident that the cavities on the photocatalyst surface have significantly decreased, which is a suitable reason for the deactivation of the employed biochar@ZnO–Ce nanocatalyst^60,61^. Since the support of this nanocatalyst is biochar derived from biomass, it is a relatively cheap catalyst with high activity. Moreover, owing to the appropriate reusability and high dye removal efficiency, it can be concluded that biochar decoration with nanoparticles can be employed as a promising approach for the photocatalytic removal of azo dyes.

## Conclusion

In this study, the performance of the photocatalytic procedure of ZnO–Ce nanoparticles decorated on biochar was investigated at LED irradiation for the removal of reactive blue 19 dye. Decoration with ZnO/Ce nanoparticles on biochar revealed a good potential to improve the photocatalytic process. The characteristics of biochar/ZnO–Ce were determined applying FE-SEM, FTIR, XRD, BET, EDX and TEM analyses. The specific surface area of biochar increased from 518.34 to 636.52 m^2^/g when decorated with ZnO-Ce nanoparticles. Moreover, under the conditions of 5 ppm dye concentration, 1 mL H_2_O_2_, 100 mg biochar/ZnO-Ce catalyst, 40 min surface adsorption and, then, under 50W LED light for 100 min and pH 9, the highest removal yield of RB 19 dye (96.47%) was achieved. The kinetic results of RB 19 dye removal revealed that the pseudo-first order kinetic model was consistent. According to the values of the correlation coefficients (R^2^), it can be confirmed that the equilibrium data are more consistent with the Sips isotherm. The addition of H_2_O_2_ leads to an in efficient elimination and lower recombination of the generated electron–hole pairs. The performance of degradation was significantly enhanced while adding H_2_O_2_. This can be attributed to the production of a large number of highly oxidative hydroxyl radicals used to degrade the organic compound. In addition, as light intensity increases, the stimulated photons lead to the generation of a large number of active radicals in water environment and better decomposition of the organic compounds. The addition of larger amounts of photocatalyst also reduces the efficiency of the process due to turbidity. Besides, the recyclability of the biochar@ZnO–Ce nanocatalyst under optimum circumstances demonstrated that the reusability of the photocatalyst exhibits only a marginal decrease of 6.08% after five cycles. This study reveals that biochar@ZnO–Ce nanocatalyst can be employed as an efficacious photocatalyst for the facilitated elimination of RB19 from an aqueous solution.

## Data Availability

All experimental data were published in the current article. The additional data and information will be provided to individuals upon official request to the corresponding authors [Mohsen Mnasouri and Zahra Noorimotlagh].
